# Structural, morphological and biological assessment of magnetic hydroxyapatite with superior hyperthermia potential for orthopedic applications

**DOI:** 10.1038/s41598-025-87111-7

**Published:** 2025-01-25

**Authors:** Smrithi Saroj, U. Vijayalakshmi

**Affiliations:** Department of Chemistry, School of Advanced Sciences, Vellore Institute of Technology, Vellore, Tamil Nadu 632014 India

**Keywords:** Iron-doped HA, Sol-gel, Cytotoxicity, Hyperthermia, Anti-bacterial activity, Biomaterials, Structural materials

## Abstract

Hydroxyapatite (HA) is an important constituent of natural bone. The properties of HA can be enhanced with the help of various ionic substitutions in the crystal lattice of HA. Iron (Fe) is a vital element present in bones and teeth. In this study, iron-doped HA was synthesized using a refluxing-based sol-gel route with varying concentrations of iron (1–9 M%). Samples were analyzed using an X-ray diffractometer (XRD), UV–Vis Spectrophotometer, Fourier-transform infrared spectroscopy (FT-IR), vibrating sample magnetometer (VSM) and Scanning Electron Microscope (SEM). The biological assessment was carried out by hemolytic assay, anti-bacterial activity and in-vitro biocompatibility. XRD data confirmed the evolution of the hexagonal HA crystal structure with the reduction in the crystallinity and the crystallite size. All the characteristic bands were confirmed using FT-IR which also further proved the existence of A-type carbonated apatite. The UV–Vis spectra confirmed the reduction in the band gap energies owing to the substitution of iron. The SEM results showed a change in the shape of the samples with increasing iron concentration. The magnetic behavior of samples also altered from diamagnetic to ferromagnetic behavior due to the doping of iron with enhanced heating efficiency. All the samples were found to be hemocompatible. The antibacterial efficacy was found to be higher for *E. coli* (gram-negative) bacteria compared to *S. aureus* (gram-positive) bacteria. Moreover, the superior cell viability of MG-63 (osteoblast-like) cells was observed in Fe-doped HA, attributed to MTT assay which revealed the enhanced cell viability of osteoblast-like cells in the Fe-doped HA. These results strongly emphasize the potential of the developed samples for bone regeneration applications.

## Introduction

The use of biomaterials has constantly evolved over the past years owing to their potential features in modern medicine. Biomaterials have found their use in various areas which include the manufacturing of dental implants, fabrication of scaffolds, as a structural function in regenerative medicine and tissue engineering^1,2^. Among the various biomaterials explored, calcium phosphates are considered to be significant with desirable features. Calcium phosphates possess unique properties such as bioactivity, biocompatibility and osteoconductivity making them potential candidates for bone substitution and bone remodeling applications. These materials also help in the proliferation and adhesion of osteoblasts (bone-forming cells) which is extremely essential for bone regeneration^3^.

There are different calcium phosphates employed for bone regeneration which mainly consist of Hydroxyapatite (HA), Tricalcium phosphate (β-TCP) and biphasic calcium phosphate (a mixture of HA and β-TCP)^4^. Among these, Hydroxyapatite (HA, Ca_10_(PO_4_)_6_(OH)_2_) is a versatile material applicable for bone tissue repair. Bone is an intricate tissue consisting of a cellular matrix that comprises collagen and other bone minerals. The mineral components are approximately 30% amorphous calcium phosphate and 70% nanocrystalline hydroxyapatite. Hence, HA has the ability to mimic the natural bone. This can also be attributed to the fact that HA has been employed for various applications related to bone tissue repair^5^. Moreover, it exhibits excellent biological properties (biocompatibility, bioactivity, osteoconductivity, etc.), along with its ability to remain stable at physiological pH, which makes it extremely suitable for orthopedic applications^6^. However, HA also has certain shortcomings limiting its potential. This mainly includes poor mechanical strength, low degradation rate thereby reducing its effect to complement in vivo bone regeneration and its comparatively poor efficiency to eliminate microbial infections while placing implants into the body^7^. Several metal-ion substitutions are possible on HA to circumvent these issues.

Iron (Fe) is an important mineral present in the body responsible for the production of hemoglobin, a protein present in red blood cells. Iron is beneficial for various biological purposes such as the synthesis of DNA, cellular respiration, transport of oxygen, gene regulation, and lipid metabolism. Many studies have reported that iron can facilitate bone regeneration. Some studies revealed that the deficiency of iron can cause hindrance to the mineralization of osteoblast cells^8^. Also, superparamagnetic nanoparticles (magnetite (Fe_3_O_4_) or maghemite (γ-Fe_2_O_3_)) can be utilized successfully for targeted drug delivery, medical imaging (MRI), and cancer therapy (magnetic hyperthermia)^9^. However, one of the major obstacles faced by iron oxide nanoparticles is the spontaneous aggregation of these particles under physiological conditions^10^. This can be overcome by developing magnetic hydroxyapatite (iron-HA or iron oxide-HA) with significant potential for several applications. Doping of iron has many advantages such as the increase in bioactivity, facilitating the osteoblast proliferation and enhancement in magnetic properties. The amount of iron plays an integral role in the pronounced behavior of magnetic HA. The changes in the concentration of iron bring remarkable differences in the lattice parameters which significantly affect the crystallite size, morphology and crystallinity. This results in the improved efficacy of magnetic HA^11^. Investigations on the bone-bonding ability of magnetic HA based on the SBF (Simulated Body Fluid) immersion studies have shown that magnetic HA has the best apatite layer formation on the surface when compared to HA^12^.

The magnetic behavior of HA changes from diamagnetic to ferromagnetic owing to the doping of iron, thereby making it a potential tool for hyperthermia applications. Magnetic hyperthermia is a cancer therapy utilized for killing cancer cells by generating heat around the tumor in the range of 42–46 °C with the presence of an alternating magnetic field. Magnetic HA helps in hyperthermia by acting as thermoseeds around the tumor, thereby causing the death of cancer cells^13^. Magnetic HA can act as a drug carrier owing to its magnetic properties and helps in targeted drug delivery. The drug release profile of magnetic HA has been studied which shows that the release of drug significantly depends on the amount of iron, making the doping of iron into HA more important in biomedical applications. The doping concentration of metal ions such as Fe, Co and Ni could enhance the heating ability of the materials. As the dopant concentration increases, the saturation magnetization increases which helps to improve the heat generation efficiency. Similarly, Singh et al. showed that a higher concentration of Cobalt in Co^2+^ doped HA exhibited superior heating efficiency^14^. There are numerous methods employed for the synthesis of magnetic HA such as coprecipitation, hydrothermal method, sol-gel, spray pyrolysis, and high-energy ball milling. Even though co-precipitation is one of the most widely used methods for the synthesis of HA, reproducibility is a serious concern due to the lack of control over the process parameters. Moreover, it requires pH-controlling agents to activate precipitation. However, the sol-gel method provides homogeneity and control of the size and shape of the particles. Furthermore, it can be carried out in ambient temperatures when compared to the hydrothermal method which requires high temperatures and expensive autoclaves for synthesis^15^.

An important drawback of using superparamagnetic iron oxides for hyperthermia is the aggregation of the iron oxide nanoparticles which severely affect its potential^16^. Iron oxide nanoparticles require surface modification to improve their stability and hydrophilicity under physiological conditions which limits their potential as hyperthermia agents. Several strategies have been utilized to improve the specific absorption rate by tailoring the size and shape of the particles and impact hyperthermia efficiency through magnetic anisotropy. However, most of the literature reports are focused on tailoring the shape of spherical iron oxide nanoparticles into cubic and rod-shaped particles to bring magnetic anisotropy into effect. Therefore, the need for developing other alternatives such as ferromagnetic materials arises. Even though several reports focused on the superparamagnetic behavior of iron-based nanomaterials, only a few research works have studied the ferromagnetic behavior of iron-doped HA synthesized using refluxing sol-gel method for hyperthermia applications. Moreover, the concentration of iron required should be optimized as a large amount of iron could be detrimental to the human body due to the toxicity produced by the leaching of iron. This can be overcome by developing iron-doped HA with tunable properties in which Fe is substituted in Ca sites without altering the structure of HA. Therefore, in this study, we have attempted the refluxing-based sol-gel synthesis of iron-doped HA and aimed to achieve a finer understanding of the structural and morphological properties, chemical composition, stability, anti-bacterial efficiency and cytotoxicity effect of the synthesized samples. The synthesized samples were characterized using XRD, FT-IR, SEM-EDS and UV-vis DRS Spectroscopy. The magnetic properties of the samples were studied using VSM. The heating efficacy of the optimized sample was carried out to analyze its hyperthermia potential. In-vitro hemocompatibility, anti-bacterial efficiency and biocompatibility of the samples were also performed to study the effect of doping of iron into the HA crystal structure for bone regeneration and other applications.

## Materials and methods

### Materials required

Ferric nitrate [Fe (NO_3_)_3_-SDFCL 98%], triethyl phosphite [P(OCH_2_CH_3_)_3_-Sigma Aldrich 98%], calcium nitrate tetrahydrate [Ca (NO_3_)_2_·4H_2_O-SDFCL 99%], double distilled water, dimethyl sulfoxide (DMSO) and phosphate buffer solution (PBS).

### Synthesis of Fe-doped hydroxyapatite

Sol-gel method based on a refluxing route was utilized to manufacture HA and Fe-HA using the precursors. For hydrolyzation of ‘P’ precursor, triethyl phosphate (0.6 M) was taken in an RB flask and refluxed at 70 °C for 8 h. The “Fe” precursor was prepared in varying concentrations such as 1, 3, 6 and 9 M% and also the “Ca” precursor was accordingly varied to achieve the stoichiometric ratio of HA,1.67 [Ca_10 − x_ Fe_x_ (PO_4_)_6_ (OH)_2_: X = 1, 3, 6 and 9 M%]. The as-prepared “Fe” and “Ca” solutions were slowly added to the above refluxed “P” precursor solution dropwise at a rate of 1 ml/min. The solution mixture which was obtained was then aged for 24 h and again refluxed at 85 °C for 16 h. This was carried out to increase the hydrolyzation of precursors and also further help to improve the reactivity of Phosphorous with Calcium and Iron. Consequently, the solution was transferred to a beaker and kept for evaporation in a water bath at 100 °C. This results in the formation of a transparent gel due to condensation. The obtained gel was drained of moisture by keeping it in a hot air oven at 100 °C for 12 h. The product was then sintered at 900 °C for 2 h. The synthesized HA was obtained with a yield of 62%. The complete hydrolyzation of the phosphorous precursor through refluxing helps to avoid the impurities that might occur during the reaction. Finally, the procured HA and Fe-HA powders were then characterized using different techniques namely UV–Vis DRS, ATR-FTIR, XRD and SEM with EDAX analysis. The magnetic properties of the samples were studied using VSM analysis. Also, HA was synthesized using the same method without adding the Fe precursor. Hereby, HA with 1, 3, 6 and 9 M % of iron are represented as HAF1, HAF3, HAF6 and HAF9 respectively.

### Characterization

The crystalline structure along with phase purity of HA and Fe-HA samples were analysed using X-ray Diffractometer (XRD, Bruker D8) with 2θ ranging from 10° to 80°. The characteristic functional groups present in HA and Fe-HA samples were determined using FT-IR with ATR mode (Shimadzu model) ranging from 4000 to 400 cm^− 1^. The UV-Vis DRS was carried out to study the band gap energies of HA and Fe-HA (JASCO, V-670 PC). The surface morphology and topography of sintered HA and Fe-HA were determined using Scanning Electron Microscopy (SEM: Carl Zeiss), and the composition of elements present in HA and Fe-HA was confirmed by Energy dispersive X-ray Analysis (EDAX). A Vibrating Sample Magnetometer (VSM) was utilized to determine the magnetic behavior of both HA and iron-doped HA. The chemical valence state of iron present in the sample was confirmed using X-ray Photoelectron Spectroscopy (XPS, ULVAC-PHI VersaProbe 4).

### Hyperthermia effect

The heating efficiency of the sample HAF6 was determined using an induction heating coil system (Easy Heat 8310, Ambrell). The heating was carried out by dispersing 10 mg of the sample in 2 ml of water and placed inside a copper induction coil (4.5 cm × 6.3 cm) which is attached to a thermocouple. An operating frequency of 335 kHz and field amplitude of 13.5 kA/m were used for the experiment. The temperature difference of the sample with time was then measured.

### In vitro hemocompatibility

Hemocompatibility assay of the synthesized samples was carried out following ASTM F756–00 guidelines, blood was drawn from a healthy adult volunteer. Before receiving blood, written informed consent was obtained from all subject involved in the study. The experimental protocols was approved by the Institutional Ethical Committee for Studies on Human subjects (IECH), and all experiments were performed in accordance with relevant guidelines and regulations of IECH (Health Center, VIT, Vellore, India: ref.no.VIT/IECH/XIII/2023/17), and the blood was collected with an anticoagulant and was further diluted with sterilized saline, where the saline was taken as negative control counter in distilled water with a positive control. The collected blood was initially centrifuged at 6000 rpm for 5 min to isolate the plasma from the red blood cells (RBC). The RBC pellet was taken and 5 ml of PBS (freshly prepared) was added to dilute it. This was again centrifuged at 6000 rpm for 5 min which was repeated with 5 ml of PBS solution 3 times. The obtained pellet was finally dissolved in 15 ml of PBS (stock solution) and stored in a refrigerator. 0.2 ml of blood is taken from the stock solution and mixed with 0.8 ml of PBS solution. The 5 mg/ml of HA and Fe-HA samples were then added to the above mixture and incubated at 37 °C for 1 h. The test samples were taken and centrifuged at 6000 rpm for 5 min. The supernatant is collected after the centrifugation and the absorbance at 545 nm is observed using a spectrophotometer. The values were taken three times to obtain reliable data. The hemolytic ratio of the samples was calculated using the following formula.

$$\text{H}\text{e}\text{m}\text{o}\text{l}\text{y}\text{t}\text{i}\text{c} \text{R}\text{a}\text{t}\text{i}\text{o} = \frac{\text{A}\text{b}\text{s}\text{o}\text{r}\text{b}\text{a}\text{n}\text{c}\text{e} \text{o}\text{f} \text{s}\text{a}\text{m}\text{p}\text{l}\text{e}-\text{A}\text{b}\text{s}\text{o}\text{r}\text{b}\text{a}\text{n}\text{c}\text{e} \text{o}\text{f} \text{n}\text{e}\text{g}\text{a}\text{t}\text{i}\text{v}\text{e}}{\text{A}\text{b}\text{s}\text{o}\text{r}\text{b}\text{a}\text{n}\text{c}\text{e} \text{o}\text{f} \text{p}\text{o}\text{s}\text{i}\text{t}\text{i}\text{v}\text{e}-\text{A}\text{b}\text{s}\text{o}\text{r}\text{b}\text{a}\text{n}\text{c}\text{e} \text{o}\text{f} \text{n}\text{e}\text{g}\text{a}\text{t}\text{i}\text{v}\text{e}}\times 100$$

### Anti-bacterial activity

The anti-bacterial efficacy of HA and Fe-HA samples were assessed using the disc diffusion method against two bacteria: a gram-positive bacterium (*S. aureus*) and a gram-negative bacterium (*E. coli*). They are the two most common microorganisms that causes infections during implantation and surgeries and hence these were selected for determining the anti-microbial efficacy of the developed samples. The medium used for the proliferation and cultivation of bacteria was Mueller-Hinton agar. The contamination of the medium was avoided by autoclaving at 120 °C for 1 h. Then the medium was transferred to petri plates which were sterilised and made to solidify for 30 min. The two different strains of fresh bacterial cultures were spread across the plates. The Whatman filter paper was used to prepare discs (5 mm diameter) and soaked into the test extract. The test extract is prepared by 5 mg/ml of 10% DMSO solution that contains HA and Fe-HA samples. The discs were then submerged on the cultured plates and incubated at 37 °C for 24 h to allow the proliferation of bacteria for further examination. Chloramphenicol antibiotic disk and DMSO solvent was taken as the positive control (PC) and negative control (NC) respectively. Finally, the bacterial repressive behaviour towards the synthesized powders were observed through zone of inhibition process and a ruler was used to measure the inhibition zone of all pathogens. The antibacterial activity was performed in triplicate to analyse the reliability of the results.

### In-vitro biocompatibility

The biocompatibility of the samples which helps to determine the cytotoxic nature was evaluated using MG-63 (National Centre for Cell Science, Pune, India) osteoblast-like cells. The osteosarcoma-derived MG-63 cell line was employed due to its osteoblast-like characteristics and its ability to produce alkaline phosphatase and osteocalcin, two important osteogenic markers that makes it the most extensively used cell line for orthopedic applications. The 96-well plate method was used to culture the human bone osteosarcoma cell line (MG-63) with an amount of 1 × 10^4^ cells/well in Dulbecco’s Modified Eagle Medium (DMEM) to enhance cell growth. The osteoblast cells were then thoroughly washed with serum-free medium and were treated with various test concentrations such as 100, 200, 300, 500, 750 ${\upmu }$g/mL of 6 and 9 M % Fe-HA. The treated cells were then incubated at 37 °C for 24 h. IC50 concentration of Doxorubicin (1 µg/mL) was taken as positive control in serum free media and incubated for 24 h. 0.5 mg/mL of MTT (3-[4,5-dimethylthiazol-2-yl]2,5-diphenyl tetrazolium bromide) prepared in PBS was added to the cells and incubated at 37 °C for 4 hr. The medium that contains MTT was removed from the cells after the incubation period and washed using PBS to discard the unreacted reagent. The formazan crystals which were formed is then dissolved with 100 µL of DMSO, the dye turns to purple blue colour and the absorbance at 570 nm is measured. Triplicate values were taken to ensure the consistency of the results.

$${\text{Percentage of cell viability}}\,=\,{\text{Sample OD}}/{\text{ Control OD }}*{\text{1}}00$$

## Results and discussion

### Powder-XRD analysis

**Fig. 1:**
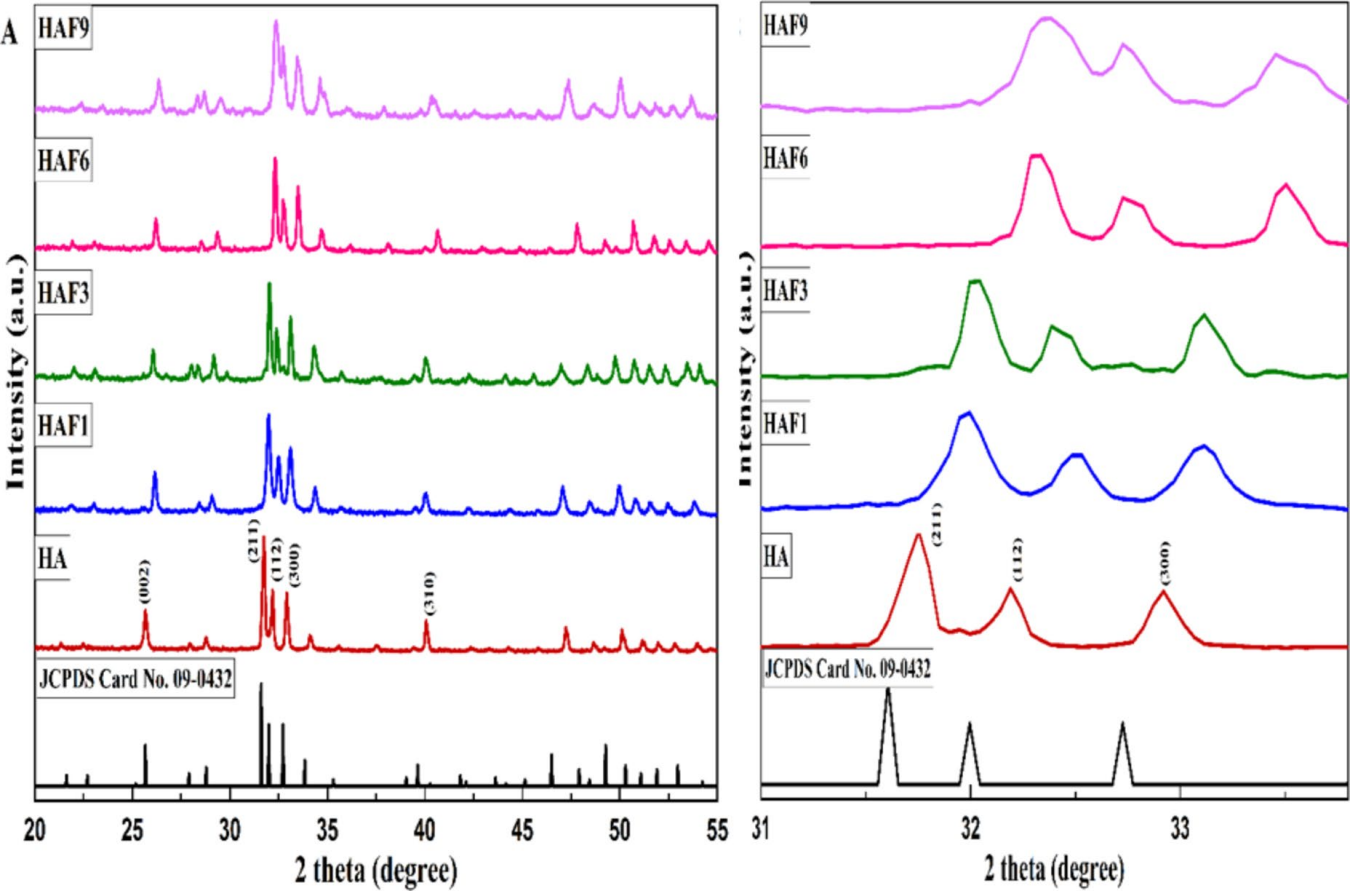
(**A**) The complete XRD spectra and (**B**) peak shift of the triplet peak observed between 31 and 33° of HA, HAF1, HAF3, HAF6 and HAF9 respectively.

The impact of iron concentration on the structure of HA was evaluated using the XRD pattern. The diffraction patterns of HA and Fe-doped HA are shown in Fig. 1(A). The XRD diagram of HA depicts the development of well-defined crystalline diffraction peaks similar to the standard hexagonal HA (JCPDS: 09-0432). The dominant peaks which were positioned at 25.88 (002), 31.63 (211) and 39.88 (310) indicated the major evidence for the formation of HA^17^. The XRD patterns of Fe-doped HA sintered at 900 °C with different concentrations (1–9 M%) can also be seen.

HA crystallizes in a hexagonal unit cell with two inequivalent Ca^2+^ sites. The Ca (I) site is attached to the phosphate group whereas the Ca (II) site is attached to the OH^−^ group and O^2−^ ions present in the angular position of PO_4_ tetrahedron. Ming Jiang et al. has reported that larger-sized and smaller-charged (e.g.: Sr^2+^) ions preferentially occupy the Ca (I) site, but the ions with small size and greater charge (Fe^3+^) tends to occupy the Ca (II) site. The atomic radius of calcium is 0.99 Å whereas that of iron is 0.64 Å. As a result, Fe^3+^ can easily replace Ca^2+^ and disturb the crystal structure of HA^18^. The crystal lattice reduction occurs due to the occupancy of a smaller ion which corresponds to the decrease in the lattice parameters as seen in Table 1. Also, it can be seen that the lattice parameter towards ‘a’ axis got increased and a gradual decline along the ‘c’ axis, which suggest the preferred growth of the crystal along the same direction. Ismat Ullah and coworkers have suggested that crystals can grow through either direction due to the difference in the lattice parameters owing to the substitution of ions^19^. These changes in the lattice parameters result in decreasing d-spacing value and hence the peaks get shifted to higher 2θ value (as obtained through Eqs. 1, 2). Z. Pasandideh et al. have reported that several factors such as ionic substitution and vacancies produce lattice distortion which thereby result in the shift of peak positions^20^. The impact of Fe concentration on the peak shift and peak broadening can be assessed in the Fig. 1B. Furthermore, the intensity of the peaks got reduced as the concentration of iron enhanced and the peaks got broadened. This corroborates to the decrease in the crystallinity of the samples due to the substitution of iron when compared to HA (Eq. 3). The dominant phase obtained after sintering at 900 °C was HA when prepared by sol-gel method which clearly shows that sintering at 900 °C does not affect its stability. During the sol-gel synthesis, the ferric ions get substituted by replacing calcium ions owing to the differences in their atomic radii and subsequently, the HA nucleation takes place, which reduces the formation of iron oxide as a secondary phase. As a result, the structural integrity of HA could be maintained even in the presence of iron.

The occupancy of a smaller ion also results in charge imbalance which can create some vacancies and could incorporate other functionalities into the structure. This led to the decrease in the crystallinity of the materials. There are two types of carbonated hydroxyapatite namely—A type and B type. When the carbonate ions get substituted for the OH^−^ ions, it is A type and when it gets substituted for PO_4_^3−^ group, it is B type^21^. It has already been reported that when it is A type, the ‘a’ axis gets elongated and the ‘c’ axis gets declined due to the inclusion of carbonate ions^22^. In iron-doped HA, it can be seen that an augmented ‘a’ axis was observed while the ‘c’ axis got a reduction as explained in Table 1. This shows that the formed apatite is an A-type carbonated apatite. The carbonated form of apatite is more beneficial for bone regeneration applications as the natural bone contains traces of carbonate and also helps in enhancing the osteoconductive potential of HA^23^.

**Table 1:** Crystallite size, crystallinity and lattice parameters of HA and Fe-HA samples.

Sample	Crystallite size {D}, nm	Lattice parameters [Å]		Crystallinity ${ ({\upchi }}_{c})$	Microstrain (ε) (×10^− 3^)
		a = b	c		
HA	52.618	9.418	6.884	1.72	1.92
HAF1	48.615	9.425	6.876	1.63	1.82
HAF3	42.806	9.438	6.870	1.42	1.63
HAF6	35.548	9.446	6.863	1.28	1.46
HAF9	28.785	9.453	6.856	0.97	1.40

The lattice parameters, crystallite size, degree of crystallinity and microstrain evaluated from the XRD data is shown in Table 1. The particle size of the synthesized HA and Fe-HA was calculated using Scherrer equation,

1 $$\text{D} =\frac{k\text{λ}}{\beta \text{cos}\theta }$$

The lattice parameters ‘a’ and ‘c’ was also calculated for HA and Fe-HA using the formula for hexagonal crystal symmetry corresponding to the crystallographic planes (002) and (300) respectively^24^,

2 $$\frac{1}{{d}^{2}}=\frac{4}{3}\left[\frac{{h}^{2}+h{k}^{2}+ {k}^{2}}{{a}^{2}}\right]+\frac{{l}^{2}}{{c}^{2}}$$

3 $${ {\upchi }}_{c}= {\left(\frac{0.24 {\text{\AA}} }{\beta }\right)}^{3}$$

4 $$\epsilon =\frac{\beta }{4 tan \theta }$$

### FT-IR analysis

**Fig. 2:**
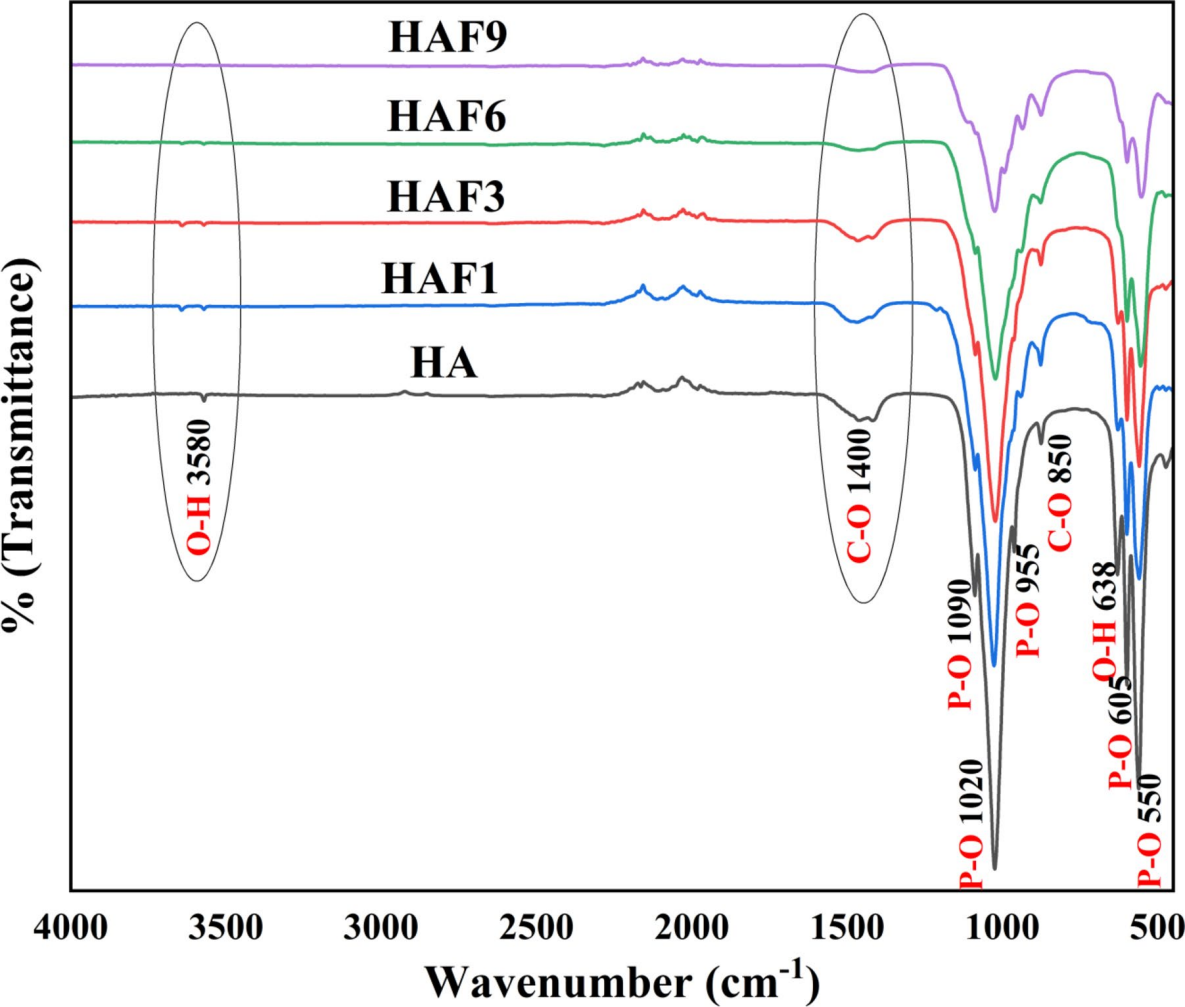
FT-IR spectra of HA and iron-doped HA.

FT-IR spectroscopy is a technique used to identify the characteristic functional groups present in and Fe-HA samples. The FT-IR spectrum of HA was depicted in Fig. 2. The peak at 550 and 605 cm^− 1^ belongs to the symmetrical bending vibrational mode of doubly degenerate υ_2_ of P–O. The hydroxyl stretching vibrational mode was observed at 638 cm^− 1^. The non-degenerate symmetrical stretching vibrational mode of P–O was found at 955 cm^− 1^. The peaks present at 1093 and 1022 cm^− 1^ belongs to the asymmetrical stretching vibrational mode of υ_3_ triply degenerate P–O bond^25^. Berzina-Cimdina et al. reported that the band present at 1033 cm^− 1^ is a characteristic peak of hexagonal HA. The sharp peak present at 3580 cm^− 1^ is attributed to the stretching vibrational mode of OH^−^ group in the HA crystal. The emergence of triplet peaks ranging between 500 and 650 cm^− 1^ and the sharp peak at 3580 cm^− 1^ are the characteristic peaks of HA, making it different from β-TCP and proving that HA is formed^26^.

The FT-IR spectra of iron doped HA also clearly notified the characteristic phosphate and hydroxyl vibrational modes for the formation of Fe-HA. The band intensity of PO_4_^3−^ at 1090–955 cm^− 1^ and 605–550 cm^− 1^ declined as the concentration of iron enhanced. This can be due to the weakening of phosphate bonds in HA as a result of the bond distortion between ions^27^. The decline in the intensity of the peaks could also be related to the combination of reduced grain growth and reduction in the crystallinity as described by Farzadi et al.^28^. Pon-on et al. explained that the incorporation of ions into HA lattice results in the reduction of stretching mode of vibration of OH group^29^. No particular peak for metal doping was seen which could be associated to the difference in the interatomic distance between the metal ions and oxygen which was explained by Laurencin et al.^30^.

The carbonate peaks were present ranging between 1350 and 1450 cm^− 1^ which relates to the asymmetric stretching vibrational mode of C–O, whereas the peak present at 850 cm^− 1^ corresponds to the bending vibrational mode of carbonate group. Previous reports suggest that these peaks arise due to the presence of absorbed CO_2_ during the synthesis. Fe^3+^ has high Lewis acid strength, thus the incorporation of Fe^3+^ results in water and CO_2_ adsorption^31^. The type of carbonated HA can also be explained using FT-IR spectra. Here it can be seen that the intensity of the carbonate peaks gets reduced with increase in the concentration of iron in the range of 1350–1450 cm^− 1^ and the intensity of the characteristic hydroxyl peak at 3580 cm^− 1^ declined significantly which therefore proposes that the apatite formed is A-type. Moreover, the human bone also contains a small amount of carbonate which indicate that carbonated HA is more beneficial when compared to stoichiometric HA. The same hypothesis is also confirmed by the XRD results. As the concentration of iron increases, the peak intensities of hydroxyl and phosphate group declines and gets broader owing to the presence of HPO_4_^2−^, which form due to the interaction between OH^−^ and PO_4_^3−^ moieties. This rectifies the charge imbalance that occur as a result of the cationic deficiency. Therefore, the overall stability is not affected significantly. The intensity of the apatitic OH^−^ group also decreased owing to dehydroxylation occurring because of the metal ions present and the same causes the decline in crystallinity of the samples as explained by Robles–Aguila et al. Moreover, as mentioned above, the formation of A-type apatite results in the replacement of OH^−^ ions by carbonate, which also leads to the decrease in the intensity of hydroxyl group^32^.

### UV–Visible DRS spectroscopy

**Fig. 3:**
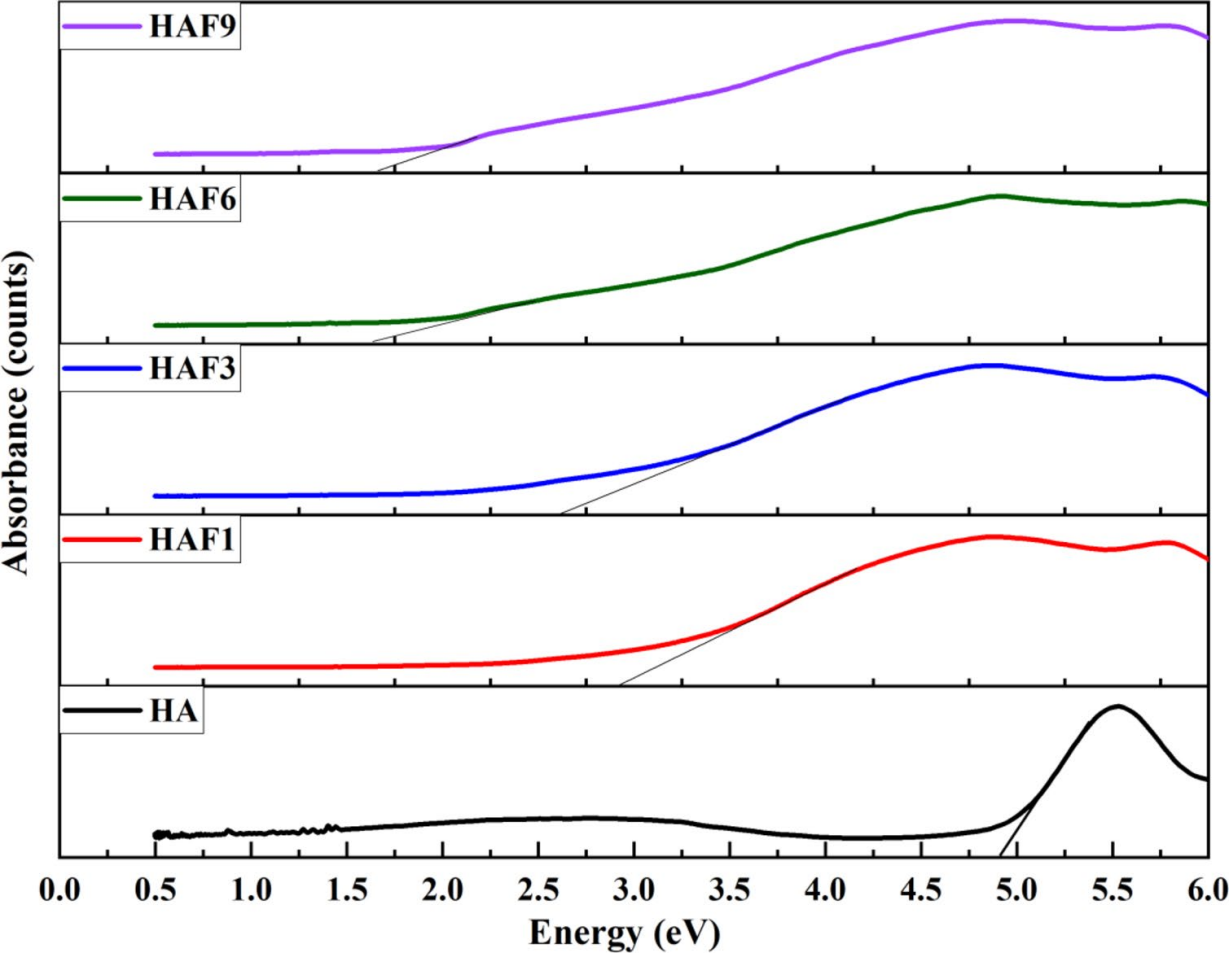
UV-Visible DRS analysis of HA and iron doped HA.

The UV–Visible DRS spectroscopy helps in depicting the electronic structure of the samples. Wood and Tauc method were utilized to determine the band gap energy of and Fe-HA from UV-Vis spectra. Figure 3 represents the DRS plot for both HA and Fe-HA. Band gap energy of 4.85 eV was obtained for HA which is similar to commercial HA (4.51–5.4 eV)^33^. The samples HAF1, HAF3, HAF6 and HAF9 also showed a band gap energy of 2.7, 2.6, 1.8 and 1.6 eV respectively. It can be stated clearly from the data that HA has higher band gap energy than Fe-HA samples owing to the incorporation of iron in HA structure. The presence of another metal ion in the lattice causes bond distortions which led to the decline in band gap energy from 4.85 to 1.6 eV. The introduction of surface and interfacial defects which develops due to the rise in the amount of iron, generates oxygen ion vacancies and hydroxyl ion vacancies in the HA lattice, thereby reducing the band gap energy which makes Fe-HA to show photocatalytic behaviour and this could be exploited for drug delivery applications^34^.

### SEM-EDAX analysis

**Fig. 4:**
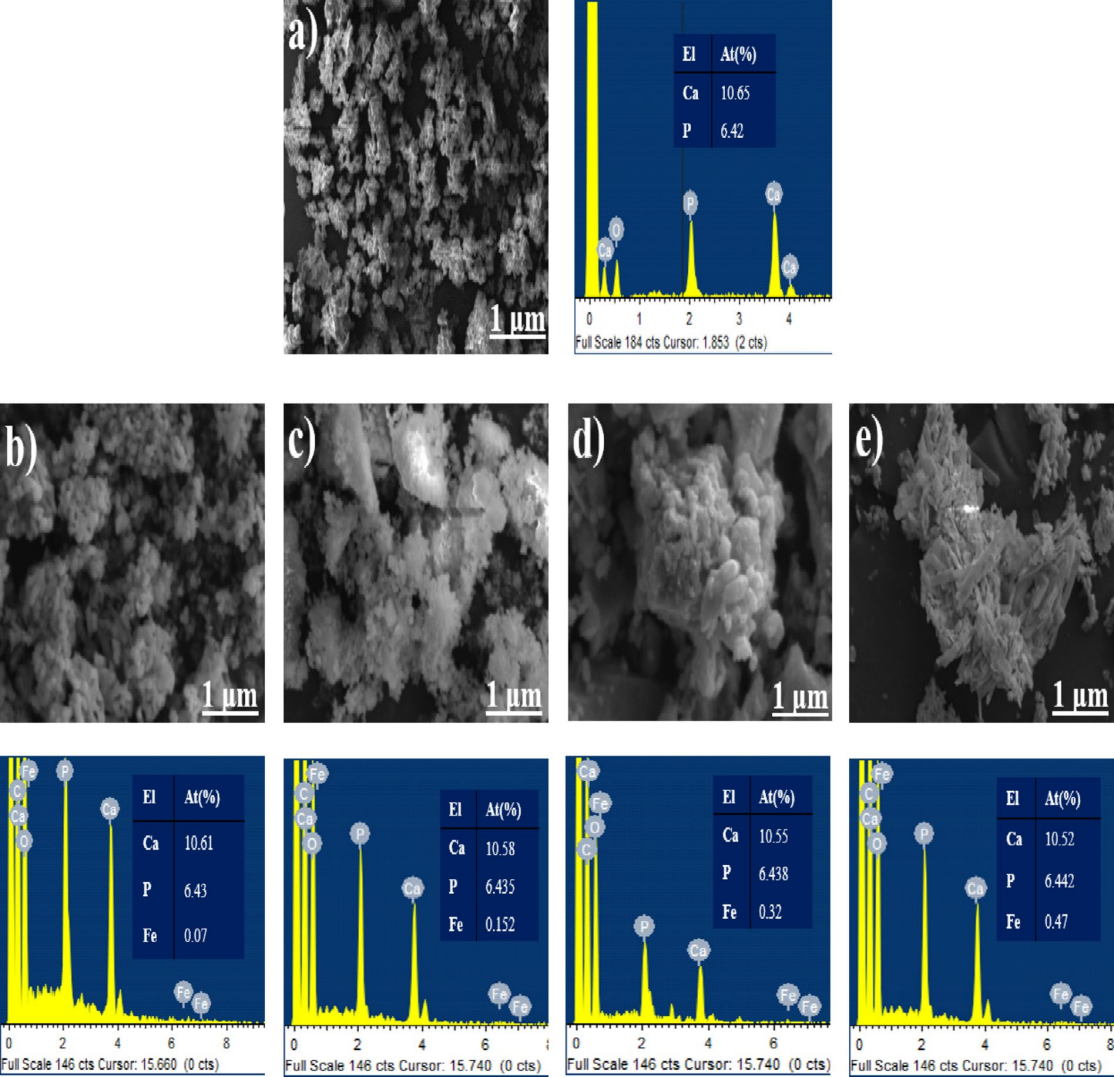
SEM-EDAX images of (**a**) HA, (**b**) HAF1, (**c**) HAF3, (**d**) HAF6 and (**e**) HAF9.

The morphological features and elemental composition of HA and Fe-HA were described in Fig. 4. The SEM image of HA (Fig. 4a) shows the presence of particles in dense nature with spherical morphology. Figure 4b–e depicts the SEM images of Fe doped HA. These images attributed to the fact that changes in the amount of iron brought drastic difference to the shape of the particles. As we move to the higher concentrations of iron doped HA, no more were the particles observed to be in spherical shape. They were found to be in flakes and more agglomerated as the amount of iron increased. Due to the augmented presence of doped ions, the growth of the crystal changes significantly from one plane to another which results in the difference in morphology of the samples as described by Tank et al.^35^. The morphology of the particles was observed to be flaky-like as the concentration of iron increased. As a result, the surface area gets enhanced which helps to improve the biological response of the material due to the presence of more active sites, making it particularly applicable for bone regeneration applications. The elemental composition was determined using the EDAX spectra. The presence of Ca and P elements in HA was corroborated using EDAX spectrum. The ratio of Ca/P was found to be 1.658, which is almost the same as that of biological apatite (1.67). In the case of Fe-HA, the presence of all the elements namely—Ca, P, Fe was identified which proves the incorporation of iron into the HA structure. The Ca + Fe/P ratios for HAF1, HAF3, HAF6 and HAF9 were 1.6540, 1.6535, 1.6525, 1.6517. This decline in the ratio is in good agreement with the XRD results which can be attributed to the reduction in the crystallinity. The same can also be related to the augment in the atomic percentage of iron, which improved from HAF1 to HAF9. There is no significant decrease in the Ca/P ratio of Fe-HA owing to the doping of iron which clearly indicates that the synthesized material is stable. The doping of iron decreases the crystallinity which in turn reduces the Ca/P ratio but does not significantly impact the stability.

### X-ray photoelectron spectroscopy (XPS) analysis

**Fig. 5:**
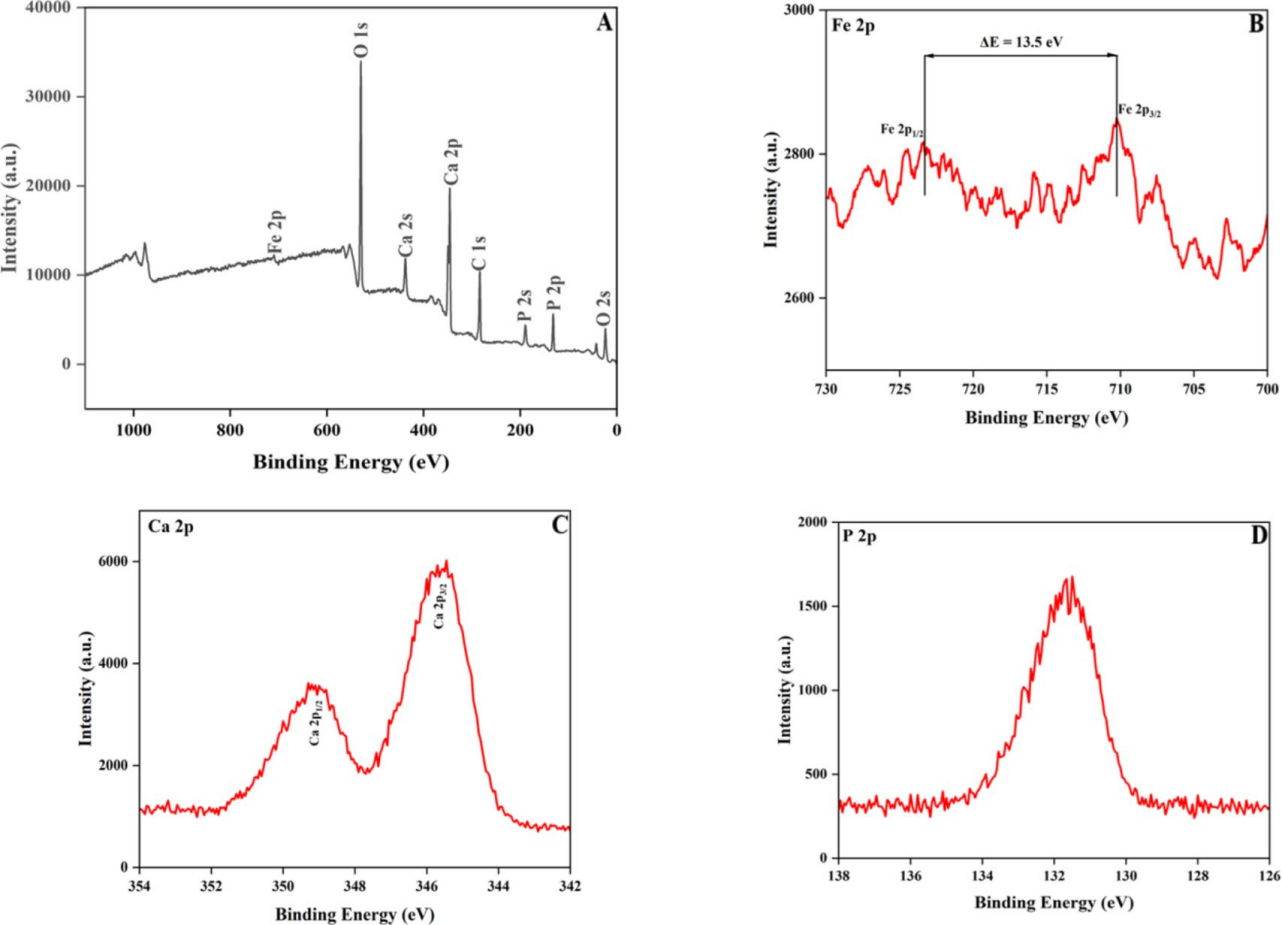
(**A**) XPS spectra of FeHA sample, HR-XPS spectra of Fe 2p (**B**), Ca 2p (**C**) and P 2p (**D**) peaks.

XPS is an important technique that provide information regarding the chemical state and composition of the elements present in the sample. The XPS spectra of 6 M% of Fe doped HA is depicted in the Fig. 5A with varying binding energy ranging between 0 and 1100 eV with reference to C 1s (285 eV). The spectrum showed the existence of elements—Ca(2p), P(2p), O(1s), Fe(2p) with binding energy of 340–350 eV, 128–136 eV, 531 eV and 705–725 eV respectively. The valence state of Ca, O and P were in accordance with the studies reported previously. The (Ca + Fe)/P ratio was found to be 1.65 which closely resembled to the stoichiometric ratio of HA (1.67), thereby suggesting that the doping of iron does not affect the stoichiometry of HA. In order to identify the oxidation state of the element present, high resolution (HR) XPS was carried out (Fig. 5B–D). The Ca 2p peak existed as a doublet peak which arises due to the spin-orbit coupling and corresponds to Ca 2p_3/2_ and Ca 2p_1/2_ respectively. The position of the peak and the energy separation between the peaks are the two integral variables which determine the oxidation state of an element. From these two parameters, ‘Ca’ was found to be existing in + 2 oxidation state that corroborates well with the elemental state of calcium present in HA. The P 2p peak consist of only one peak which point out that the chemical environment around P is identical and the peak position belongs to the phosphate group as explained by Alizadeh et al.^36^.

The oxidation state of iron was determined using HR XPS spectrum. Two major peaks were observed at around 710 and 723 eV which correlates to Fe 2p_3/2_ and Fe 2p_1/2_ respectively. The existence of these peaks indicate that iron is present in + 3 oxidation state. The energy separation between these two peaks was found to be 13.5 eV which further prove the + 3-oxidation state of iron^37^. Therefore, XPS results evidenced the successful doping of Fe^3+^ ions into the crystal lattice structure of HA.

### VSM analysis

**Fig. 6:**
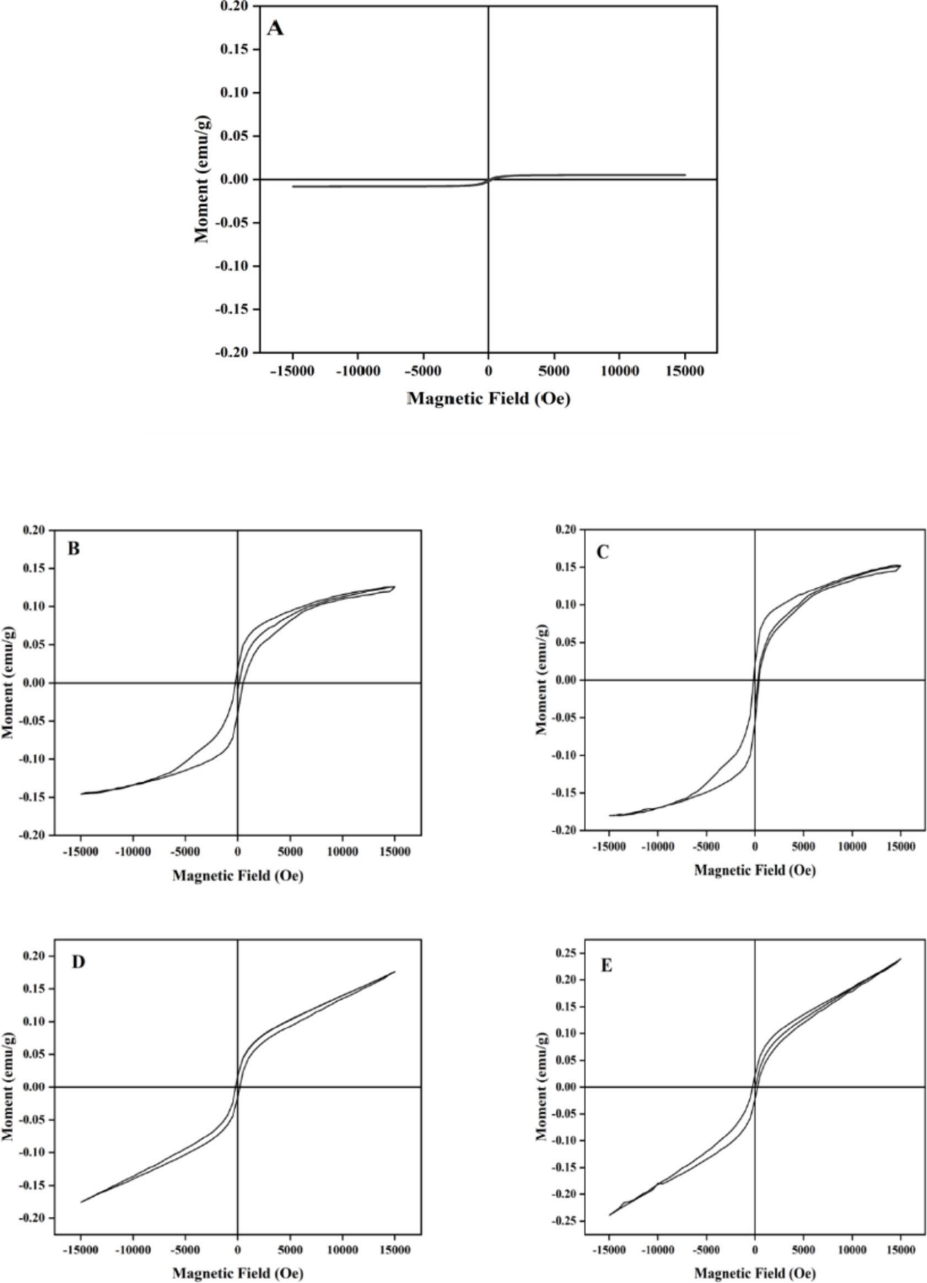
VSM analysis of HA (**A**), HAF1 (**B**), HAF3 (**C**), HAF6 (**D**), HAF9 (**E**) samples.

VSM Analysis is carried out to determine the magnetic behaviour of Fe-HA. The results depicted in Fig. 6 shows the significant difference on the magnetic behaviour of HA and iron doped HA. The saturation magnetization of the samples is depicted in Table 2. This works on the principle of Faraday’s law of induction where a change in electric field will cause an enhancement in the magnetic field and further the changes in magnetic field is measured using VSM^38^. All the doped samples exhibit a hysteresis loop which is indicative of the ferromagnetic property of the materials. In the case of HA, the magnetic moment is parallel to the applied field which indicates the diamagnetic property of HA. When iron gets incorporated into HA, the formation of a hysteresis loop occurs which makes them ferromagnetic in nature. For lower concentrations of iron, the loop was not symmetric owing to the non-uniform distribution of iron in the lattice. As the concentration increases, the loop becomes for symmetric which denotes perfect distribution of iron^39^. In iron doped HA, the inclusion of both Fe^2+^ and Fe^3+^ results in anisotropic lattice contraction due to the charge imbalance between Ca and Fe and forms a covalent bond with the oxygen present in phosphorous or hydroxyl bond which gets stabilised at higher temperatures and account for its magnetic behaviour. Saturation Magnetisation also enhanced with the amount of iron which is indicative of ferromagnetic property. Thus, the study proves that the prepared materials show strong intrinsic magnetic properties which can be exploited for drug delivery and hyperthermia applications.

**Table 2:** Saturation magnetisation of HA and Fe-HA samples.

Sample	Magnetization (emu/g)
HA	0.005
HAF1	0.125
HAF3	0.152
HAF6	0.176
HAF9	0.248

### In-vitro hemocompatibility

**Fig. 7:**
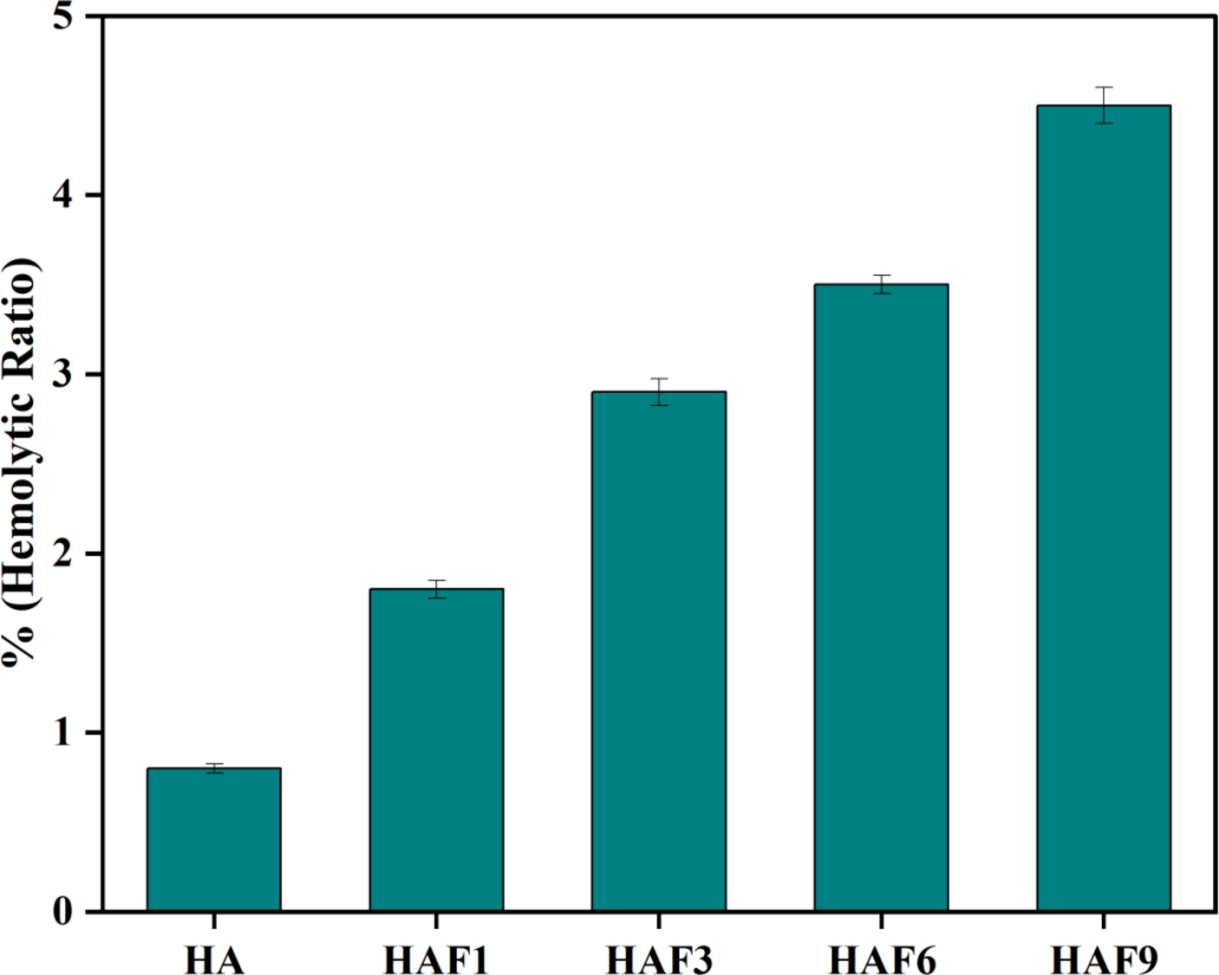
Hemolytic ratio of and iron doped HA.

The blood compatible behaviour of the samples could be examined using hemocompatibility study. According to the ASTM: 756–00 guidelines, the ratio of hemolysis is divided into three categories such as > 5%, 2–5%, < 2% which is classified as hemolytic, slightly hemolytic and non-hemolytic respectively^40^. The ratio of hemolysis for HA and Fe-HA were shown in Fig. 7. The hemolytic ratio was found to be steadily increasing with varying concentrations of Fe doped HA. The hemolytic ratio of HA and HAF1 was observed to be 0.8 and 1.8% which were highly hemolytic. As the concentration of iron increased, there was a rise in hemolytic ratio. The hemolytic ratio of HAF3, HAF6, and HAF9 was shown to be 2.9, 3.5 and 4.5% respectively. This indicate that with the increase in the amount of iron, the samples become slightly hemolytic. The crystallinity of the samples gets reduced due to the incorporation of iron which further results in the resorption and leaching out of Fe^3+^ ions, which makes the samples more hemolytic. These ions interact with the RBCs, resulting in the rupture of the membrane and hemoglobin gets liberated from the erythrocytes, which accounts for the higher degree of hemolysis^41^. This data proves that the developed samples have the hemolytic ratio > 5% and therefore can be utilized for various applications.

### Anti-bacterial activity

**Fig. 8:**
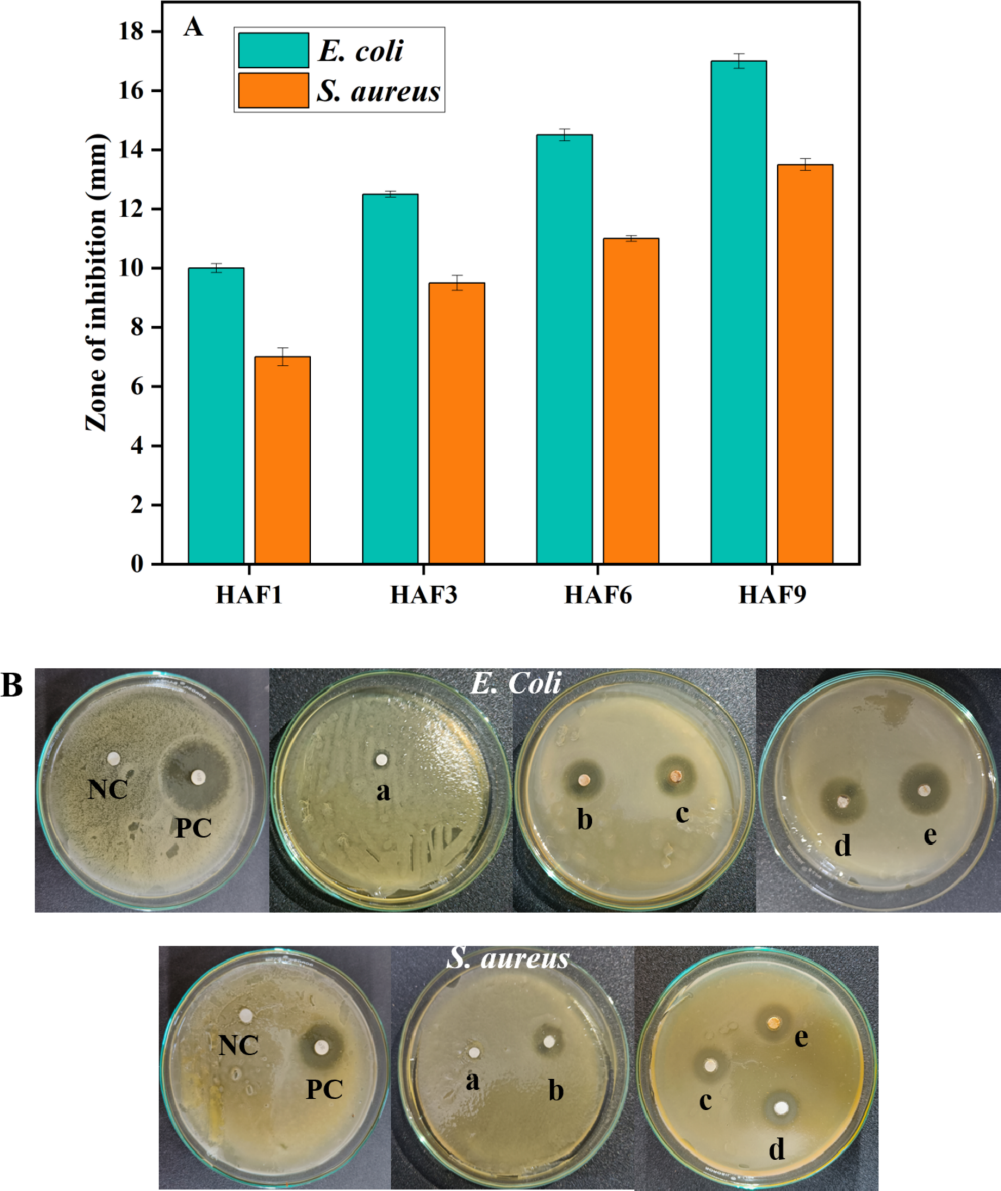
(**A**) Zone of inhibition of iron doped HA. (**B**) Disk diffusion method to determine the anti-bacterial activity of (a) HA, (b) HAF1, (c) HAF3, (d) HAF6 and (e) HAF9 samples respectively.

Disk diffusion method was employed to determine the microbial restriction efficacy of the doped samples. The inhibition zone of the microbes was estimated and represented in Fig. 8A, B. It was observed that the bacterial growth declined as the concentration of iron increased. The inhibitory effect for *E. coli* was observed to be superior and the values were found to be 3, 10, 12.5, 14.5 and 17 mm whereas the values for *S. aureus* were 1, 7, 9.5, 11 and 13.5 mm for HA, HAF1, HAF3, HAF6 and HAF9 respectively. The highest zone of inhibition was recorded for chloramphenicol antibiotic, while the DMSO solvent showed no inhibition zone which implies that it does not have any effect on bacterial cultures. The inhibition zone for *E. coli* was higher than *S. aureus* due to the difference in the cell wall thickness between gram negative and gram positive bacteria^42^. The cell wall of *E. coli* consists of peptidoglycans and polysaccharides and is comparatively thin. However, *S. aureus* has a thick cell wall which contains murein, mucopeptides and lipoteichoic acids^43^. This makes the cell wall to be less permeable for ions to enter inside. Also, the presence of antioxidant enzymes (catalase) in *S. aureus* provides the bacteria with higher resistance against foreign ions. All these factors lead to the higher susceptibility of gram negative bacteria towards antibiotics than gram positive bacteria.

The incorporation of a metal ion (Fe) reduces the crystallinity of the material which lead to sedate resorption of Fe-HA in DMSO. This results in the association of released ions with the cell wall of microorganisms. When the samples get uniformly dispersed in DMSO, positively charged Fe^3+^ ions get released from the structure owing to the reduced crystallinity. Then these ions interact with the functional groups present in the structural proteins of the cell membranes and enhances its permeability which attributes to the discharge of intracellular constituents and finally causes the death of bacteria. The lower crystallinity for HAF6 and HAF9 contributes to higher release of iron and hence the zone of inhibition observed is more. The discharged ions help in the production of active oxygen species which causes damage to the cell membrane of the bacteria. The ions which get released get attached to the DNA and prevents its replication and cell proliferation. Finally, this will conduce to the death of bacteria^44^.

### Hyperthermia effect

Though, HAF9 exhibited higher saturation magnetization, considering the toxic nature of the material at higher concentration of iron as explained through hemolysis, HAF6 was considered to be the best fit for hyperthermia studies.

**Fig. 9:**
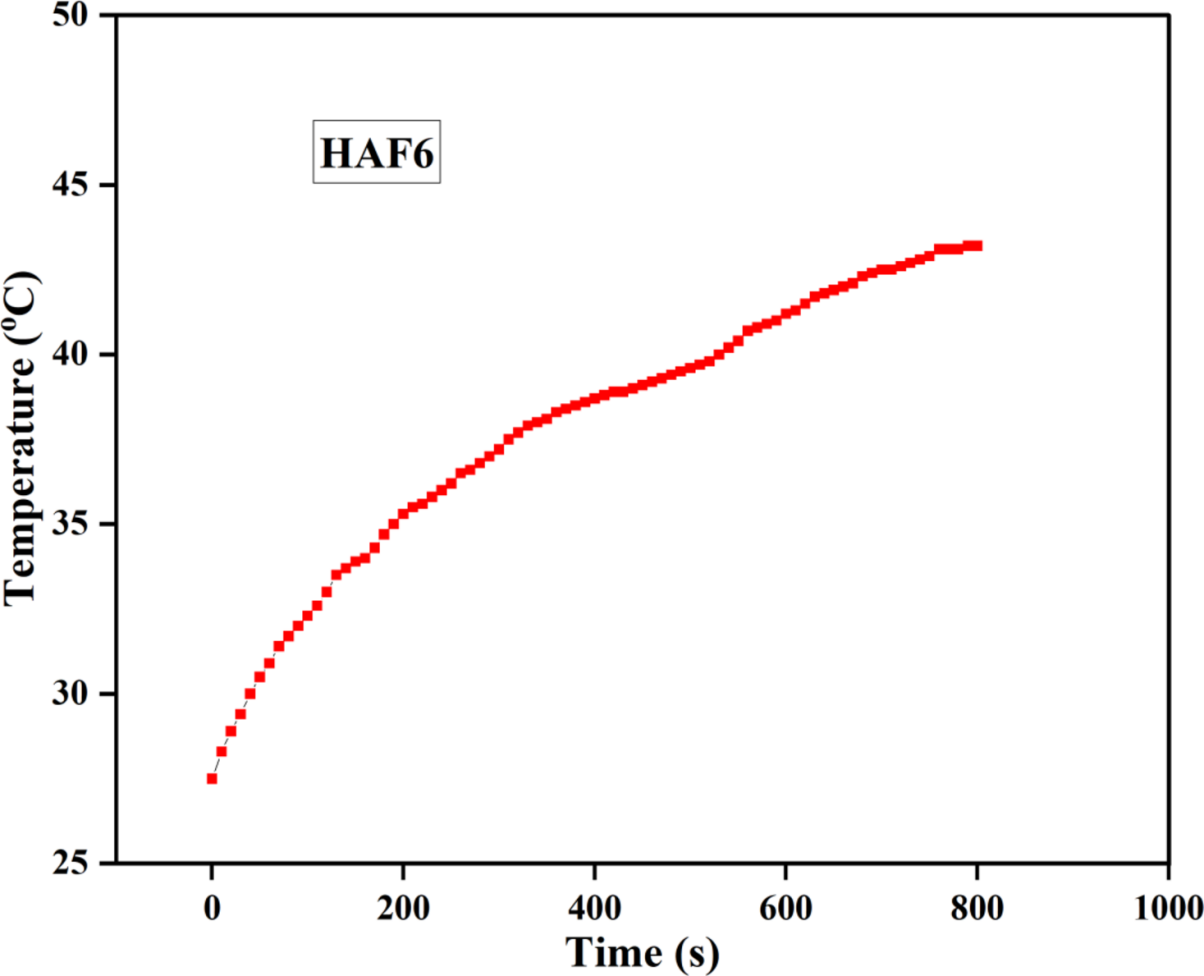
Heating efficiency of sample HAF6 measured by hyperthermia treatment.

The heating efficiency of the sample HAF6 was depicted in Fig. 9. The findings show that the sample could achieve 42 °C by 660 s. The optimal hyperthermia temperature required to initiate cancer apoptosis is 42–45 °C. For ferromagnetic materials, heat is generated through hysteresis losses whereas Neel and Brownian relaxation play a role in producing heat for superparamagnetic materials^45^. Ferromagnetic materials have huge magnetic anisotropic effect which helps in generating heat through hysteresis loss. However, the impact of Brownian motion gets limited as the synthesized material gets attached to the cell walls and its movement gets reduced in viscous medium because cancerous tissue is highly viscous. This weakens the potential to attain the hyperthermia limit. Hence, ferromagnetic materials are increasingly exploited for hyperthermia applications when compared to superparamagnetic materials. Since HA does not possess any magnetic behaviour, the saturation magnetisation values of the samples are less after doping of iron and similar results were observed by Sarathchandra et al. Therefore, the time required to reach hyperthermia limit was also higher when compared to other superparamagnetic materials. Even though the saturation magnetization is lower for ferromagnetic materials than superparamagnetic materials, there are other factors such as concentration, applied magnetic field, frequency and anisotropic effect which are responsible for achieving hyperthermia temperature^46^. The normal cells do not get affected by increasing temperature as they have the ability to dissipate the heat generated unlike tumorous cells. This is because of the reduced blood flow in the cancerous cells due to the dysfunctional behavior of the blood vessels. As a result, heat gets entrapped in the tumor cells and finally leads to cell death.

The specific absorption rate (SAR)of the sample was evaluated using the following equation:

4 $$\text{S}\text{A}\text{R}\hspace{0.17em}=\hspace{0.17em}\text{C} \frac{\varDelta T}{\varDelta t}\frac{1}{m}(\text{W}/\text{g})$$

where C is the specific heat capacity of the solvent used (water—4.186 J g^− 1^ K^− 1^), m is the mass fraction of the material used, ΔT/Δt is the initial slope of the time-temperature curve. The SAR of the sample was found to be 22.6 W/g. Higher SAR value is necessary and advantageous for hyperthermia treatment. Though, iron oxide-HA composites have superior SAR values, the aggregation of iron oxide is a challenge inhibiting its potential for in-vivo applications^47^. Therefore, magnetic HA can be utilized without compromising its ability for hyperthermia. However, a detailed study is required for optimizing sample concentration, frequency and field strength of the synthesized material to examine the hyperthermia properties.

### In-vitro cytotoxicity study

**Fig. 10:**
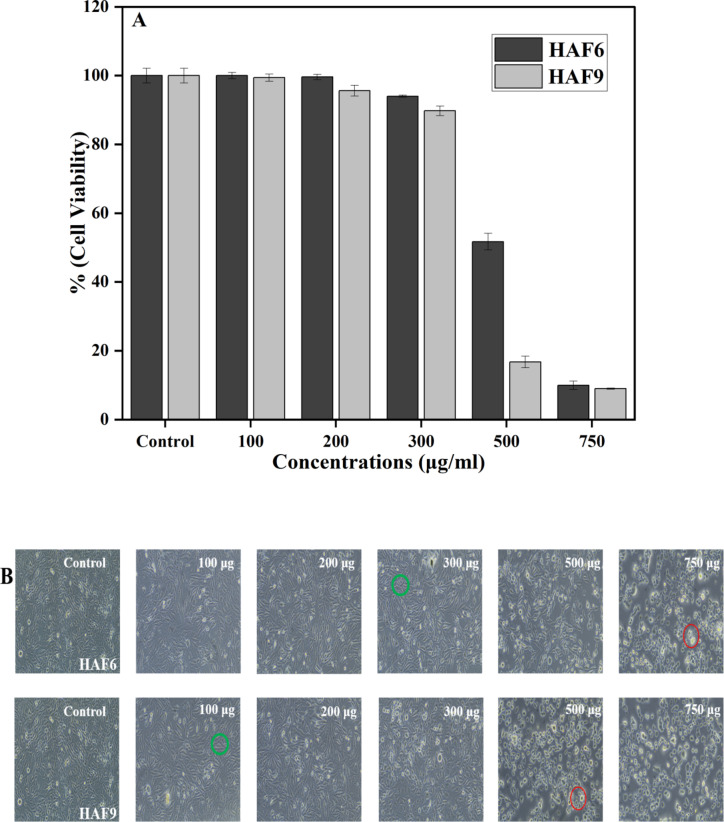
(**A**) Percentage of cell viability of HAF6 and HAF9 samples. (**B**) Microscopic images of MG-63 cells at 100–750 µg/mL incubated for 24 h. (green and red circles represent the live and dead cells respectively.

The HAF6 and HAF9 samples were taken to investigate the cytocompatibility and cell viability behaviour using MG-63 cell line at different ratios ranging from 100 to 1000 µg/ml using MTT Assay. The cell viability (%) of the samples were shown in Fig. 10(A). The results evidenced the fact that MG-63 cells were highly compatible and metabolically active with in the range of 100–300 µg/ml concentrations of HAF6. But when the concentration of iron increases to 9 M%, the cell viability greatly decreased for 500 µg/ml when compared to 6 M%. This clearly indicates that it is toxic to cells at higher concentration of iron. Osteoblast like MG-63 cells show good cellular response at lower concentrations, while at higher concentrations, the cellular response is poor owing to the discharge of Fe^3+^ ions and causes toxicity. When iron is incubated with cells, it oxidizes intracellular proteins thereby producing oxidizing agents which results in the decreased cell viability^48^. The microscopic images (Fig. 10(B)) displayed the interaction of MG-63 cells which shows the cellular attachment, spreading, proliferation capacity up to 300 µg/ml. The live and the dead cells were denoted using green and red circles respectively. The cells exhibited polygonal morphology with good adhesion onto the surface of Fe-HA powder. At higher concentration, cell death occurs which is shown by the change in the morphology to spherical shape. Previous reports suggest that improved cell viability and proliferation in the Fe doped HA was observed when compared to clinically used hydroxyapatite NPs^49^. The doped ion can have an extensive impact on the absorption of proteins and other biomolecules. It can also help to improve the cell attachment and proliferation as emphasized by Lu et al.^50^. Therefore, these results prove that doping of iron helps in cellular attachment along with improved osteoblast cell metabolism.

## Conclusions

In this study, Fe-doped HA was prepared by a refluxing based sol-gel method which is a more advantageous method as pH controlling agents and gelling agents are not required and the obtained powder was sintered at 900 °C for further studies. Doping led to a decrease in the crystallinity as well as lattice parameters as depicted by XRD pattern due to the replacement of Ca^2+^ ion by a smaller Fe^3+^ ion in HA. The band gap energy determined using UV–visible DRS spectroscopy decreased from 4.85 to 1.6 eV owing to the formation of surface and interfacial defects in Fe doped HA. The addition of iron can also impart ferromagnetic behaviour to the material as showed by VSM analysis which can be exploited for magnetic hyperthermia. The sample was able to reach 42 °C by around 6 min and could help in achieving cellular apoptosis of tumor. The rate of hemolysis of the developed samples were less than 5% and accounted for the excessive hemocompatible nature. Higher concentrations of iron doped HA with lower crystallinity displayed significant inhibitory effect against *E. coli* than *S. aureus*. The biocompatibility study of the powders showed good cell viability up to concentration of 300 µg/ml but higher concentrations (500 and 750 µg/ml) exhibited toxicity due to the discharge of Fe^3+^ ions from Fe-HA. Microscopic observations displayed healthy cell attachment and growth at both the concentrations.

To conclude, these in-vitro studies evidenced that the synthesized Fe doped HA shows ability as a potential bone substitute material, providing desirable properties suitable for various biomedical uses including targeted drug delivery and magnetic hyperthermia.

## Data Availability

The authors declare that the data supporting the findings of this study are available within the paper. Should any raw data files be needed in another format they are available from the corresponding author upon reasonable request.As the corresponding author Dr.U. Vijayalakshmi can be contacted for Data Availability.

## References

[1] Ding, B. et al. A strong and tough hydroxyapatite-based fiber with enamel-inspired hierarchical structure. *Sci. China Mater.* **67**, 2496–2504 (2024).

[2] Tang, G. et al. Recent trends in the development of bone regenerative biomaterials. *Front. Cell. Dev. Biol.* **9**, 665813 (2021). 34026758 10.3389/fcell.2021.665813 PMC8138062

[3] Sawada, M., Sridhar, K., Kanda, Y. & Yamanaka, S. Pure hydroxyapatite synthesis originating from amorphous calcium carbonate. *Sci. Rep.* **11**, 11546 (2021). 34078994 10.1038/s41598-021-91064-y PMC8173018

[4] Sun, Y., Wang, Y., Ji, C., Ma, J. & He, B. The impact of hydroxyapatite crystal structures and protein interactions on bone’s mechanical properties. *Sci. Rep.* **14**, 9786 (2024). 38684921 10.1038/s41598-024-60701-7 PMC11059379

[5] Yang, J., Li, Q., Feng, Y. & Zeng, Y. Iron deficiency and iron deficiency anemia: potential risk factors in bone loss. *Int. J. Mol. Sci.* **24**, 6891 (2023). 37108056 10.3390/ijms24086891 PMC10138976

[6] Priyadarshini, B., Stango, A. X., Balasubramanian, M. & Vijayalakshmi, U. In situ fabrication of cerium-incorporated hydroxyapatite/magnetite nanocomposite coatings with bone regeneration and osteosarcoma potential. *Nanoscale Adv.* **5**, 5054–5076 (2023). 37705779 10.1039/d3na00235g PMC10496897

[7] Abdelmoaty, A. & Mousa, S. Synthesis and characterization of hydroxyapatite nanoparticles from calcium hydroxide fouled with gases evolved from smokestack of glass industry. *Sci. Rep.* **14**, 10969 (2024). 38745040 10.1038/s41598-024-60970-2 PMC11094126

[8] Avakyan, L. et al. Iron in hydroxyapatite: interstitial or substitution sites? *Nanomaterials* **11**, 2978 (2021). 34835742 10.3390/nano11112978 PMC8625999

[9] Sitthisang, S. et al. Nanomechanical mapping of PLA hydroxyapatite composite scaffolds links surface homogeneity to stem cell differentiation. *Sci. Rep.* **14**, 21097 (2024). 39256445 10.1038/s41598-024-72073-z PMC11387746

[10] Baskaran, P., Udduttula, A. & Uthirapathy, V. Development and characterisation of novel Ce-doped hydroxyapatite–Fe3 O4 nanocomposites and their in vitro biological evaluations for biomedical applications. *IET Nanobiotechnol.* **12**, 138–146 (2018).

[11] Chang, D. et al. Biologically targeted magnetic hyperthermia: potential and limitations. *Front. Pharmacol.* **9**, 831 (2018). 30116191 10.3389/fphar.2018.00831 PMC6083434

[12] Borciani, G. et al. Osteoblast and osteoclast activity on collagen-based 3D printed scaffolds enriched with strontium-doped bioactive glasses and hydroxyapatite nanorods for bone tissue engineering. *Biomed. Mater.* **19**, 065007 (2024). 10.1088/1748-605X/ad72c3 39173660

[13] Li, H. et al. Preparation and properties of carbon nanotube (Fe)/hydroxyapatite composite as magnetic targeted drug delivery carrier. *Mater. Sci. Eng. C* **97**, 222–229 (2019). 10.1016/j.msec.2018.11.042 30678906

[14] Singh, R. P., Singh, D. & Singh, J. P. Biomagnetic mesoporous nanorods for hyperthermia, drug delivery, and tissue regeneration applications. *J. Sol-Gel Sci. Technol.* **97**, 155–166 (2021).

[15] Wu, Y., Chen, G. & Zhu, P. Convenient synthesis of hydroxyapatite-coated ferroferric oxide microspheres by hydrothermal method. *Mater. Lett.* **253**, 218–221 (2019).

[16] Lamkhao, S. et al. Synthesis of hydroxyapatite with antibacterial properties using a microwave-assisted combustion method. *Sci. Rep.* **9**, 4015 (2019). 30850662 10.1038/s41598-019-40488-8 PMC6408465

[17] Priyadarshini, B., Anjaneyulu, U. & Vijayalakshmi, U. Preparation and characterization of sol-gel derived ce 4 + doped hydroxyapatite and its in vitro biological evaluations for orthopedic applications. *Mater. Des.* **119**, 446–455 (2017).

[18] Aksakal, B. et al. The influence of cold rolling and hydroxyapatite coating on the mechanostructure, corrosion resistance, cell viability, and antibacterial activity of ZnCu biodegradable implants. *J. Mater. Res.* **1**, 1–15. 10.1557/s43578-024-01340-6 (2024).

[19] Ullah, I. et al. Simultaneous co-substitution of Sr2+/Fe3 + in hydroxyapatite nanoparticles for potential biomedical applications. *Ceram. Int.* **44**, 21338–21348 (2018).

[20] Pasandideh, Z., Tajabadi, M., Javadpour, J. & Mirkazemi, S. M. The effects of Fe3 + and Co2 + substitution in Ca10-x-yFexCoy (PO4)6 (OH)2 hydroxyapatite nanoparticles: magnetic, antibacterial, and improved drug release behavior. *Ceram. Int.* **46**, 16104–16118 (2020).

[21] Vijayalakshmi, U. & Rajeswari, S. Influence of process parameters on the sol–gel synthesis of nano hydroxyapatite using various phosphorus precursors. *J. Sol-Gel Sci. Technol.* **63**, 45–55 (2012).

[22] Aksakal, B., Isın, E., Aslan, N., Cihangir, S. & Sezek, S. Influence of plastic deformation and hydroxyapatite coating on structure, mechanical, corrosion, antibacterial and cell viability properties of zinc based biodegradable alloys. *Met. Mater. Int.* 1–18. 10.1007/s12540-024-01710-z (2024).

[23] Wei, L., Yang, H., Hong, J., He, Z. & Deng, C. Synthesis and structure properties of Se and Sr co-doped hydroxyapatite and their biocompatibility. *J. Mater. Sci.* **54**, 2514–2525 (2019).

[24] Safarzadeh, M., Chee, C. F., Ramesh, S. & Fauzi, M. A. Effect of sintering temperature on the morphology, crystallinity and mechanical properties of carbonated hydroxyapatite (CHA). *Ceram. Int.* **46**, 26784–26789 (2020).

[25] Trzaskowska, M., Vivcharenko, V. & Przekora, A. The impact of hydroxyapatite sintering temperature on its microstructural, mechanical, and biological properties. *Int. J. Mol. Sci.* **24**, 5083 (2023). 36982158 10.3390/ijms24065083 PMC10049015

[26] Berzina-Cimdina, L. & Borodajenko, N. In *Infrared Spectroscopy-Materials Science, Engineering and Technology*, 251–263 (InTech, 2012).

[27] Kothapalli, C., Wei, M., Vasiliev, A. & Shaw, M. T. Influence of temperature and concentration on the sintering behavior and mechanical properties of hydroxyapatite. *Acta Mater.* **52**, 5655–5663 (2004).

[28] Farzadi, A., Bakhshi, F., Solati-Hashjin, M. & Asadi-Eydivand, M. & Abu Osman, N. A. Magnesium incorporated hydroxyapatite: synthesis and structural properties characterization. *Ceram. Int.* **40**, 6021–6029 (2014).

[29] Pon-On, W. et al. Physicochemical and biochemical properties of iron-loaded silicon substituted hydroxyapatite (FeSiHAp). *Mater. Chem. Phys.* **141**, 850–860 (2013).

[30] Laurencin, D. et al. Magnesium incorporation into hydroxyapatite. *Biomaterials* **32**, 1826–1837 (2011). 21144581 10.1016/j.biomaterials.2010.11.017

[31] Veerla, S. C., Kim, J., Sohn, H. & Yang, S. Y. Controlled nanoparticle synthesis of Ag/Fe co-doped hydroxyapatite system for cancer cell treatment. *Mater. Sci. Eng. C* **98**, 311–323 (2019). 10.1016/j.msec.2018.12.148 30813033

[32] Robles-Águila, M. J., Reyes-Avendaño, J. A. & Mendoza, M. E. Structural analysis of metal-doped (Mn, Fe, Co, Ni, Cu, Zn) calcium hydroxyapatite synthetized by a sol-gel microwave-assisted method. *Ceram. Int.* **43**, 12705–12709 (2017).

[33] Kumar, B. K. S., Jagannatham, M., Venkateswarlu, B., Dumpala, R. & Sunil, B. R. Synthesis, characterization, and antimicrobial properties of strontium-substituted hydroxyapatite. *J. Aust. Ceram. Soc.* **57**, 195–204 (2021).

[34] Hadagalli, K. et al. Effect of Fe3 + substitution on the structural modification and band structure modulated UV absorption of hydroxyapatite. *Int. J. Appl. Ceram. Technol.* **18**, 332–344 (2021).

[35] Tank, K. P., Chudasama, K. S., Thaker, V. S. & Joshi, M. J. Pure and zinc doped nano-hydroxyapatite: synthesis, characterization, antimicrobial and hemolytic studies. *J. Cryst. Growth* **401**, 474–479 (2014).

[36] Alizadeh, N. & Salimi, A. Facile synthesis of Fe-doped hydroxyapatite nanoparticles from waste coal ash: fabrication of a portable sensor for the sensitive and selective colorimetric detection of hydrogen sulfide. *ACS Omega* **7**, 42865–42871 (2022). 36467963 10.1021/acsomega.2c04905 PMC9713890

[37] Carrera, K. et al. Formation of vacancy point-defects in hydroxyapatite nanobelts by selective incorporation of Fe3 + ions in ca (II) sites. A CL and XPS study. *Mater. Sci. Eng. B* **271**, 115308 (2021).

[38] Iannotti, V. et al. Fe-doping-induced magnetism in nano-hydroxyapatites. *Inorg. Chem.* **56**, 4446–4458 (2017). 10.1021/acs.inorgchem.6b03143 28379709

[39] Bakhshi, V., Poursadegh, H., Amini-Fazl, M. S., Salari, D. & Javanbakht, S. Synthesis and characterization of bio-nanocomposite hydrogel beads based on magnetic hydroxyapatite and chitosan: a pH-sensitive drug delivery system for potential implantable anticancer platform. *Polym. Bull.* **81**, 7499–7518 (2024).

[40] Prodana, M. et al. A new complex ceramic coating with carbon nanotubes, hydroxyapatite and TiO2 nanotubes on Ti surface for biomedical applications. *Ceram. Int.* **41**, 6318–6325 (2015).

[41] Ma, Z., Bai, J., Wang, Y. & Jiang, X. Impact of shape and pore size of mesoporous silica nanoparticles on serum protein adsorption and RBCs hemolysis. *ACS Appl. Mater. Interfaces* **6**, 2431–2438 (2014). 24460090 10.1021/am404860q

[42] Ciobanu, C. S., Iconaru, S. L., Le Coustumer, P., Constantin, L. V. & Predoi, D. Antibacterial activity of silver-doped hydroxyapatite nanoparticles against gram-positive and gram-negative bacteria. *Nanoscale Res. Lett.* **7**, 1–9 (2012). 22721352 10.1186/1556-276X-7-324 PMC3422172

[43] Ragab, H. S. et al. Synthesis and in vitro antibacterial properties of hydroxyapatite nanoparticles. *IOSR J. Pharm. Biol. Sci.* **9**, 77–85 (2014).

[44] Tejaswini, T., Keerthana, M., Vidyavathi, M. & Kumar, R. S. Design and evaluation of atorvastatin-loaded chitosan-hydroxyapatite composite bioscaffolds for wound-healing activity. *Futur. J. Pharm. Sci.* **6**, 1–14 (2020).

[45] Duraisamy, K. et al. Fabrication of multifunctional drug loaded magnetic phase supported calcium phosphate nanoparticle for local hyperthermia combined drug delivery and antibacterial activity. *ACS Appl. Bio Mater.* **6**, 104–116 (2022). 36511628 10.1021/acsabm.2c00768

[46] Sarath Chandra, V. et al. Blood compatibility of iron-doped nanosize hydroxyapatite and its drug release. *ACS Appl. Mater. Interfaces* **4**, 1200–1210 (2012). 22316071 10.1021/am300140q

[47] Srinivasan, B. et al. Thermally modified iron-inserted calcium phosphate for magnetic hyperthermia in an acceptable alternating magnetic field. *J. Phys. Chem. B* **123**, 5506–5513 (2019). 31244102 10.1021/acs.jpcb.9b03015

[48] Vignesh, P., Ramanathan, S., Ashokkumar, M. & Ananthi, V. Biodegradable Mg–3Zn alloy/titanium–hydroxyapatite hybrid composites: corrosion and cytotoxicity evaluation for orthopedic implant applications. *Trans. Indian Inst. Met.* **1**, 1–10. 10.1007/s12666-024-03281-4 (2024).

[49] Tampieri, A. et al. Intrinsic magnetism and hyperthermia in bioactive Fe-doped hydroxyapatite. *Acta Biomater.* **8**, 843–851 (2012). 22005331 10.1016/j.actbio.2011.09.032

[50] Lu, J. et al. Preparation and preliminary cytocompatibility of magnesium doped apatite cement with degradability for bone regeneration. *J. Mater. Sci. Mater. Med.* **22**, 607–615 (2011). 21258847 10.1007/s10856-011-4228-4

